# Acute basilar artery occlusion with recurrent shivering: A case report

**DOI:** 10.1097/MD.0000000000022451

**Published:** 2020-09-25

**Authors:** Chan-Hyuk Lee, Seung-Ho Jeon, Sang Yeon Kim, Byoung-Soo Shin, Hyun Goo Kang

**Affiliations:** a Department of Neurology and Research, Institute of Clinical Medicine of Jeonbuk National University; b Biomedical Research Institute, Jeonbuk National University Medical School and Hospital, 20 Geonji-ro, Deokjin-gu, Jeonju, South Korea.

**Keywords:** basilar artery, hypothalamus, ischemic stroke, shivering, thermoregulation

## Abstract

**Rationale:**

Shivering is an important physiological response of the body that causes muscle tremors to maintain temperature homeostasis. Traumatic brain injuries that affect the hypothalamus cause hypothermia, and physical removal of suprasellar tumors causes thermoregulation imbalance. However, no study has reported shivering due to ischemic stroke.

**Patient concerns:**

A 58-year-old male patient was admitted to our emergency department to evaluate severe stenosis of the basilar artery. While waiting for further examination, he exhibited coarse shivering and severe dysarthria.

**Diagnosis:**

Brain computed tomography angiography revealed occlusion of the entire basilar artery, and cerebral hypoperfusion was diagnosed in that area.

**Interventions:**

Transfemoral cerebral angiography (TFCA) was immediately performed, followed by thrombectomy of the basilar artery.

**Outcomes:**

Neurological deficits, including shivering, were rapidly reversed. The same symptom reoccurred 5 hours later, and TFCA was performed for thrombectomy and stenting, and neurological symptoms immediately reversed. The patient's neurological symptoms did not worsen during hospitalization.

**Lessons:**

Patients with acute basilar artery occlusion need prompt management because they have a higher mortality rate than those with other intracranial artery occlusions. When a patient exhibits neurological deficits accompanied by abrupt shivering for no specific reason, basilar artery occlusion must be considered.

## 1 Introduction

The basilar artery is an important major cerebral artery that supplies blood to key structures of the limbic system, including the brainstem, thalamus, and hypothalamus. These structures have centers that control essential functions for maintenance of life. Basilar artery stenosis or occlusion causes severe neurological deficits, and the prognosis is generally poor.^[1]^ Therefore, it is critical to recognize when neurological deficits in a patient are caused by disintegration of the basilar artery. A traumatic injury^[2]^ involving the hypothalamus causes hypothermia, and physical removal of suprasellar tumors causes an imbalance in thermoregulation.^[3,4]^ However, an association between shivering and acute ischemic stroke has not been reported. We report the case of a patient who recurrently experienced reversible shivering caused by basilar artery occlusion.

## 2 Case report

A 58-year-old male patient visited our emergency department with acutely worsened non-whirling type dizziness, which first occurred 7 days prior. It lasted approximately 30 minutes and disappeared. Previously, he was diagnosed with vertebrobasilar insufficiency (VBI) due to severe basilar artery stenosis confirmed during brain magnetic resonance imaging (MRI) and angiography, which were performed at a local hospital 7 days prior. He was diagnosed as having hypertension and hypothyroidism and was on medication. He had been taking aspirin 100 mg and atorvastatin 20 mg daily. Results of neurological examinations, which included cranial nerve and cerebellar function tests, were unremarkable.

**Figure 1:**
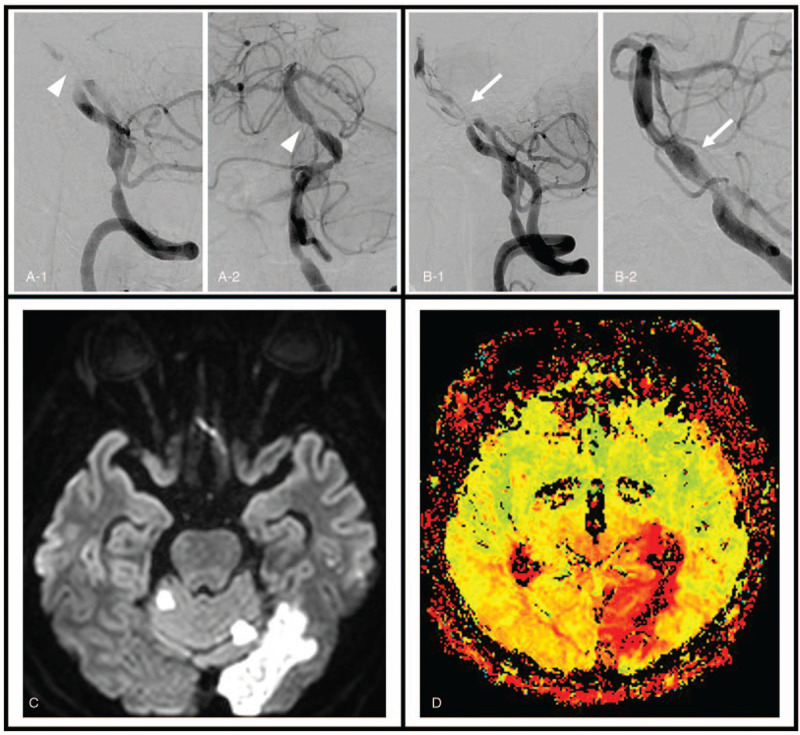
Images from a 58-year-old male patient with basilar artery occlusion. (A) This is a conventional angiography image acquired immediately after acute ischemic stroke. Severe stenosis with filling defects were seen in the mid-basilar artery on basilar artery angiography (A-1, arrow head). Post-thrombectomy angiography showing recanalization of the basilar artery (A-2, arrow head). (B) This is a conventional angiography image acquired after the second ischemic attack. Reocclusion of the mid-basilar artery was confirmed (B-1, arrow), and the endovascular stent was placed after mechanical thrombectomy. Post-stenting conventional angiography showing successful recanalization of the basilar artery (B-2, arrow). (C) Brain magnetic resonance (MR) images taken after stent insertion. Diffusion-weighted image showing diffusion restriction in both superior cerebellar artery and left posterior cerebral artery territory, but the brainstem remains intact. (D) In MR perfusion imaging, the time to maximum map showing delayed perfusion in the basilar artery region, including the hypothalamus and left occipital area.

Results of routine laboratory examinations, blood chemistry, and coagulation test were normal, but triglyceride levels increased to 350 mg/dL. Thyroid function test showed that thyroid-stimulating hormone level decreased to 0.151 μIU/mL and free T4 level increased to 24.07 pmol/L. Electrocardiogram showed a normal sinus rhythm. Cardiac output was 58% on transthoracic echocardiography. In addition, there was no cardiomegaly or regional wall motion abnormality. The patient experienced dizziness for 7 days, which suddenly worsened on the day of visit. Therefore, computed tomography (CT) perfusion was planned to obtain additional information to confirm VBI. However, while waiting for the test, he exhibited shivering accompanied by sudden chilling sensation. Neurological examination revealed drowsiness, severe dysarthria, and clumsiness and numbness in both forearms. His blood pressure was 150/100 mm Hg, body temperature was 37.2°C, and blood glucose level was 137 mg/dL. Brain CT perfusion with angiography showed basilar artery occlusion and decreased perfusion in the affected area (Fig. 1A). He was diagnosed with acute ischemic stroke due to basilar artery occlusion. Intravenous tissue plasminogen activator was administered, and endovascular intervention was performed. Stent insertion was challenging due to the high tortuosity of the basilar artery. Therefore, only aspiration thrombectomy was performed using the penumbra system (Penumbra, CA). The vessel was fully recanalized (Thrombolysis in cerebral infarction scale 3), and his symptoms resolved completely.

He was under observation in the neurology intensive care unit for 5 hours. Shivering with chilling reoccurred, and his consciousness worsened to a deep drowsy state. Spontaneous downbeat nystagmus and severe dysarthria were confirmed in a cranial nerve examination. Left-sided muscle power was reduced to medical research council grade 4+. Brain CT angiography showed basilar artery reocclusion. Therefore, intra-arterial thrombectomy was performed again, followed by endovascular stent placement (Fig. 1B), resulting in a diagnosis of vertigo, right homonymous hemianopsia, and left sensorineural hearing impairment. However, shivering, dysarthria, and mental deterioration disappeared immediately after the procedure.

He underwent brain diffusion-weighted MRI, which confirmed acute ischemic stroke in the superior cerebellar artery region of the cerebellum and posterior cerebral artery region of the right occipital lobe (Fig. 1C). The MR perfusion image showed hypoperfusion in the hypothalamus and left occipital areas (Fig. 1D). Finally, he was diagnosed with multifocal infarction in the basilar artery region due to basilar artery occlusion. He received aspirin 100 mg, clopidogrel 75 mg, and atorvastatin 40 mg once a day.

## 3 Discussion

We report the case of a patient who visited our hospital for dizziness caused by basilar artery stenosis. His neurological symptoms, possibly induced by basilar artery occlusion, aggravated in the emergency room. He was also shivering. When the occluded basilar artery was recanalized using intra-arterial thrombectomy, his shivering improved. His neurological symptoms accompanied by shivering worsened again due to basilar artery reocclusion. His symptoms improved after basilar artery recanalization and stent placement.

Shivering induces muscle tremor in response to low body temperature. It is an important physiological response to maintain constant body temperature.^[5]^ The hypothalamus regulates body temperature by collecting and integrating information about temperature from various organs and responding appropriately. The hypothalamus is a collection of nuclei responsible for maintaining body homeostasis and largely comprises anterior, tuberal, and posterior regions.^[6]^ Preoptic and anterior hypothalamic nuclei in the anterior region and the posterior nucleus in the posterior region are associated with body temperature regulation.

Thermal receptors present on the skin and some internal organs detect coldness and warmness, and electrical signals are transmitted along the lateral spinal tract or trigeminal nerve. Heat and cold-sensitive neurons are distributed in the preoptic nucleus, and the response of the posterior nucleus is determined by its relative signal intensity.^[7]^ Because the posterior nucleus contains temperature-insensitive interneurons, it functions like a thermostat. Thus, posterior nucleus has an independent set-point for body temperature, which it compares to the signal transmitted from the preoptic nucleus.^[8]^ When the transmitted signal is higher than the set-point, it activates mechanisms for lowering body temperature (e.g., vasodilation and sweating), whereas when the signal is lower than the set-point, it activates mechanisms for increasing body temperature (e.g., shivering and vasoconstriction). The thermal set-point varies depending on various internal and external conditions. Physiologically, body temperature fluctuates diurnally and is the lowest during sleep or early in the morning. However, it is approximately 1°C higher in the evening.^[9]^ Body temperature of women varies during the menstrual cycle and is approximately 1°C higher than the usual temperature in the luteal phase. The set-point can also be changed by external conditions. Pyrogens, such as endotoxins, increase the set-point by lowering the activity of heat-sensitive neurons. Although the mechanism is poorly understood, the set-point might increase in the presence of a space-occupying lesion or during dehydration.^[7]^

Arterial blood is mainly supplied to the anterior region, including the preoptic nucleus, by the anterior communicating artery (anterior circulation) and perforating branch of the anterior cerebral artery. The posterior nucleus is supplied by branches of the thalamoperforating artery that originates from the site immediately after the basilar artery branches to the posterior cerebral artery.^[10]^ Therefore, if basilar artery stenosis progresses further in these patients, the posterior hypothalamus, supplied by the basilar artery, is likely to be in a hypoperfusion state.

**Figure 2:**
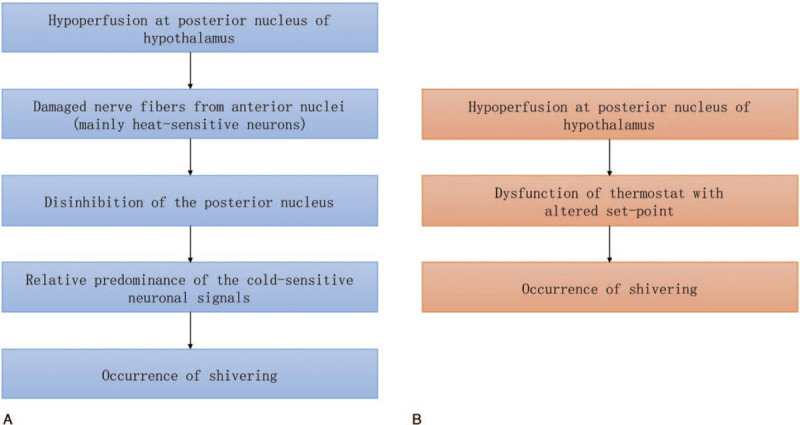
Mechanism of shivering due to hypoperfusion of the posterior hypothalamus. (A) First hypothesis. (B) Second hypothesis.

Two possible mechanisms can cause shivering due to hypoperfusion of the posterior hypothalamus. One mechanism is dysfunction of the anterior nucleus fibers. These fibers pass through the posterior hypothalamic nuclei. Therefore, if posterior nuclei are damaged, fibers of the adjacent anterior nucleus may also be damaged. Specifically, if heat-sensitive neurons are mainly damaged, signals from the cold-sensitive neurons become relatively more prevalent. As a result, posterior nuclei activate mechanisms for increasing body temperature, thereby causing shivering (Fig. 2A). Normally, there are 4 times more heat-sensitive neurons than cold-sensitive neurons, implying that heat-sensitive neurons are more likely to be damaged when exposed to an ischemic event.^[11]^ The other possible mechanism is posterior nuclei dysfunction. When hypoperfusion of posterior nuclei occurs due to basilar artery occlusion, they may lose their thermostat function or become unstable, which can cause fluctuations in the set-point, leading to shivering (Fig. 2B).

Previously, associations between hypothalamic damage and body temperature regulation have been reported. In a study involving 8 patients who underwent suprasellar resection, sympathetic nerve response to elevated body temperature was reduced compared with that in normal subjects.^[4]^ In another study, patients with traumatic brain injuries involving the hypothalamus exhibited periodic hypothermia.^[2]^ In these studies, patients did not shiver. However, in our study, the patient shivered as a result of ischemia. We suspect that shivering was not observed in the previous studies because functions of the hypothalamus, including the set-point function, were completely lost due to trauma or resection.

In a study on body temperature and stroke, hypothermia in patients with ischemic stroke had a slightly lower threshold for vasoconstriction and shivering than that in normal subjects.^[12]^ However, to our knowledge, no study has reported shivering associated with hypoperfusion in the basilar artery region. It is possible that shivering was ignored because many cases of basilar artery-related stroke exhibited severe neurological deficits including decreased consciousness. In our case, the entire basilar artery was occluded, and shivering occurred as soon as neurologic symptoms worsened. In addition, the symptoms immediately disappeared after intra-arterial thrombectomy, suggesting a causal relationship between hypoperfusion in the basilar artery region and shivering.

Basilar artery occlusion damages the brain area essential for survival. Therefore, the mortality rate is higher for basilar artery occlusion than for occlusion in other parts of the brain, and survivors are more likely to have severe complications. Therefore, rapid management is required. When a patient admitted to an emergency department suddenly shows neurological deficits accompanied by shivering, ischemia in the basilar artery region should be considered.

## Author contributions

CHL, SHJ, and HGK participated the design of this research. SHJ, SYK, and BSS collected and analyzed the raw clinical data. CHL, SHJ, BSS, and HGK carried out computational studies and wrote the manuscript. All authors have read and approved the final manuscript.

**Conceptualization:** Chan-Hyuk Lee, Sang Yeon Kim, Byoung-Soo Shin, Hyun Goo Kang.

**Data curation:** Chan-Hyuk Lee, Seung-Ho Jeon.

**Formal analysis:** Seung-Ho Jeon, Byoung-Soo Shin.

**Funding acquisition:** Hyun Goo Kang.

**Investigation:** Chan-Hyuk Lee.

**Methodology:** Chan-Hyuk Lee, Seung-Ho Jeon, Sang Yeon Kim, Hyun Goo Kang.

**Supervision:** Sang Yeon Kim, Byoung-Soo Shin, Hyun Goo Kang.

**Validation:** Byoung-Soo Shin.

**Visualization:** Hyun Goo Kang.

**Writing – original draft:** Chan-Hyuk Lee, Seung-Ho Jeon.

**Writing – review & editing:** Hyun Goo Kang.
